# Selected Parameters of the Mental State of Women with Endometriosis—A Systematic Review

**DOI:** 10.3390/jcm15103598

**Published:** 2026-05-08

**Authors:** Damian Zieliński, Kamila Tokarczyk, Magdalena Piegza

**Affiliations:** 1 Student Scientific Club, Department of Psychiatry, Faculty of Medical Sciences in Zabrze, Medical University of Silesia in Katowice, 42-612 Tarnowskie Góry, Poland; 2 Department of Psychiatry, Faculty of Medical Sciences in Zabrze, Medical University of Silesia in Katowice, 42-612 Tarnowskie Góry, Poland

**Keywords:** endometriosis, depression, anxiety, chronic pelvic pain, quality of life, mental health, sleep disturbances, sexual dysfunction

## Abstract

**Background**: Endometriosis is a chronic, estrogen-dependent inflammatory disorder affecting millions of women worldwide and constitutes a major cause of chronic pelvic pain and reduced quality of life. Increasing evidence suggests that the burden of the disease extends beyond physical symptoms and includes substantial psychological distress. This systematic review aimed to summarize current evidence regarding the association between endometriosis and mental health outcomes, including depression, anxiety, sleep disturbances, sexual dysfunction, and quality of life. **Methods**: A systematic literature search was conducted in PubMed, Europe PMC, and Google Scholar to identify studies published between 2015 and 2025 evaluating psychological outcomes in women diagnosed with endometriosis. Studies were screened according to predefined inclusion criteria and analyzed qualitatively. A total of 93 studies were included in the final review. **Results**: Women with endometriosis consistently demonstrated higher prevalence and severity of depressive symptoms and anxiety compared with control populations. Health-related quality of life was significantly reduced across studies. Chronic pelvic pain, particularly its intensity, persistence, and neuropathic-like characteristics, emerged as a key factor mediating the relationship between endometriosis and psychological distress. Additional contributors included sleep disturbances, fatigue, impaired sexual functioning, reduced self-esteem, and relationship difficulties. Emerging evidence also suggests that chronic inflammation, neuroimmune interactions, and gut–brain axis dysregulation may contribute to the coexistence of endometriosis and mood disorders. **Conclusions**: Endometriosis is associated with adverse mental health outcomes and reduced quality of life. These findings highlight the importance of multidisciplinary management strategies integrating pain control, psychological assessment, and psychosocial interventions to improve overall patient well-being.

## 1. Introduction

Endometriosis is an estrogen-dependent disorder characterized by the presence of endometrial-like tissue outside the uterus. They can also line the peritoneal cavity, and deeply infiltrating lesions form in the pouch of Douglas [1]. In rare cases, it may also affect distant organs such as the lungs or brain [2]. It is characterized by chronic pain, autonomic disturbances such as bloating, nausea, fatigue, and, in some cases, may lead to fertility problems [3,4]. Endometriosis is a heterogeneous disease with three distinct phenotypes: superficial peritoneal endometrial lesions, ovarian endometrial cysts, and deep endometriotic lesions. A specific form of endometriosis, adenomyosis, can also be distinguished, in which lesions are located within the uterine muscle itself [5].

According to data collected by the World Health Organization (WHO), it is estimated that approximately 190 million women of reproductive age worldwide suffer from endometriosis (EM), making it the second most common gynecological disease. It is most often diagnosed in women aged 25 to 29, but the diagnostic process remains significantly delayed—the average time from the appearance of the first symptoms to diagnosis is approximately 10.4 years in Germany and 4.4 years in the United States [6].

In Poland, endometriosis poses a significant public health challenge. It is estimated that approximately one million women are affected by the disease, with the number of undiagnosed cases possibly reaching three million. Given the prevalence of this condition, delays in diagnosis, and limited access to adequate treatment, a new model for organizing and financing care for patients with deep infiltrating endometriosis was introduced on 1 July 2025. Medical facilities have been designated for diagnosis, procedures, surgeries, specialist consultations, and disease monitoring, with the costs covered by the National Health Fund [7]. The new reimbursement program offers hope for faster and better multi-specialty care—diagnostics, treatment, post-operative care, psychological support, and physiotherapy.

Endometriosis is associated not only with somatic symptoms, but also with social, economic and psychological consequences, often unnoticeable in the first phase of the disease [8]. Chronic pelvic pain can significantly reduce quality of life, lead to physical exhaustion, and contribute to the development of mental health problems, including depression and anxiety disorders [9]. For some patients, the severity of symptoms significantly impedes their professional functioning and education. Furthermore, pain can negatively impact intimate relationships and the sexual health of partners. Low public awareness of the symptoms and complications of endometriosis contributes to the downplaying of early signs of the disease, further delaying diagnosis and limiting the possibility of implementing effective treatment [10]. Growing evidence also suggests a complex relationship between endometriosis and psychological well-being. Chronic pain may mediate the development of mental disorders, and recent genetic studies suggest common mechanisms predisposing to the development of endometriosis and some mental disorders [11]. Understanding the symptoms of endometriosis and their impact on patients’ daily functioning can provide the basis for developing an integrated therapeutic approach, encompassing tools that support psychological coping with the disease and provide patients with essential information about their health [12].

This article provides a comprehensive review of the medical literature on endometriosis and its relationship to mental health, sexual health, and its social determinants. It also discusses possible interventions that could potentially improve the well-being of patients.

## 2. Materials and Methods

The review was conducted according to the PRISMA 2020 (Preferred Reporting Items for Systematic Reviews and Meta-Analyses guidelines). The process included defining eligibility criteria, a systematic database search, independent review of publications by two authors, and full-text analysis of selected articles. The review was not registered with PROSPERO. The PRISMA checklist is available in Supplementary Table S2.

Studies (2015–2025) on endometriosis and selected mental health parameters were included, including those examining depression, anxiety, quality of life, psychological effects of the disease, or sexual satisfaction in women with a confirmed diagnosis (clinically, surgically, or using questionnaires). Full-text publications in Polish or English of an observational nature were included, clinical trials, meta-analyses and systematic reviews. Extracted data included study design, sample size, population characteristics, and assessed mental health outcomes.

Studies were excluded if they lacked full text, had a sample size of <20, had no clearly defined purpose, involved animals, were conference abstracts, commentaries, letters, or protocols. Additionally, studies were excluded if they included patients with other chronic diseases or infertility, assessed only surgical outcomes without psychological analyses, and lacked key terms (depression, anxiety, mental health, endometriosis) or made the results difficult to interpret.

A systematic literature search was conducted using PubMed (National Library of Medicine, Bethesda, MD, USA), Europe PMC (European Bioinformatics Institute, Hinxton, UK), and Google Scholar (Google LLC, Mountain View, CA, USA). The last search was conducted in December 2025. Data were extracted independently by two reviewers using a standardized data extraction form. Combinations of keywords and “AND” and “OR” terms were used, including “endometriosis,” “depression,” “anxiety,” “mental health,” “quality of life,” “sexual satisfaction,” and “psychological impact.” Search terms were based on both free-form phrases and MeSH terms. A detailed search strategy including combinations of keywords and MeSH terms was applied, and search strings are available upon request.

**Figure 1:**
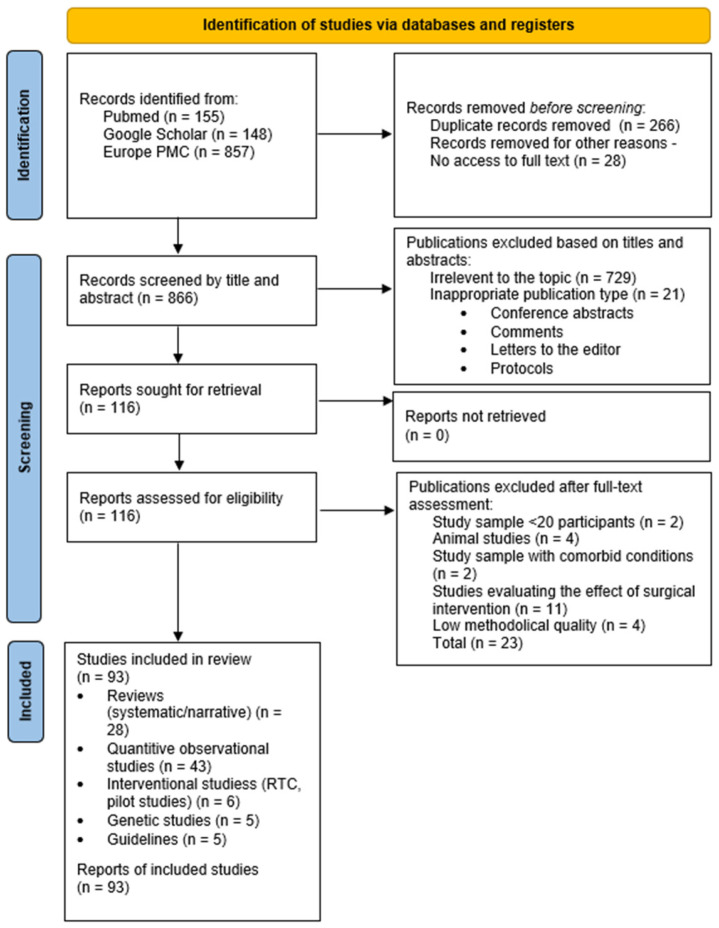
Scheme of selection of scientific publications that were included in the article. PRISMA flow diagram.

A total of 1160 records were identified across all databases. After removing duplicates and articles without full-text access, 866 papers remained. A qualitative synthesis of the included studies was performed. After an initial review of titles and abstracts, 116 articles were eligible for full-text review. Ultimately, 93 publications were included. The searches and initial reviews were conducted independently by two authors. The data extracted from the included studies were organized and summarized in a table presenting the key characteristics of each study, enabling comparison. The analysis was conducted narratively by grouping the results into thematic categories corresponding to the main research topics and presenting them in separate subsections. Due to the heterogeneity of the studies, no meta-analysis was performed, and a descriptive synthesis was applied to identify key trends and discrepancies. Results were stratified according to study design (observational studies vs. systematic reviews and meta-analyses) to avoid duplication of evidence. The methodological quality of included observational studies was assessed using the Newcastle–Ottawa Scale (NOS) which was conducted using Microsoft Excel (Microsoft Corporation, Redmond, WA, USA). The scale evaluates three domains: selection of study groups, comparability of groups and ascertainment of exposure or outcomes. Chronic pelvic pain was considered the most important confounding factor. NOS assessment was applied only to observational non-randomized studies; cross-sectional studies were assessed using the Adapted Newcastle-Ottawa Scale. Secondary sources and randomized/interventional studies were not assessed with NOS. The entire selection process is presented in a flowchart compliant with PRISMA 2020. The tables included in the manuscript were prepared using Microsoft Word (Microsoft Corporation, Redmond, WA, USA). The detailed flowchart for the selection process is provided in Figure 1.

## 3. Results

The majority of included studies were observational, while a smaller proportion consisted of systematic reviews and meta-analyses. Findings from observational studies formed the primary basis of interpretation, whereas results from reviews were used to support and contextualize the observed associations.

Among the 93 records, 19 observational studies were assessed using NOS (summarized in Supplementary Table S1), 36 cross-sectional studies were assessed using adapted NOS (summarized in Supplementary Table S2) and 38 records were not assessed because NOS was not appropriate for their design. Of the assessed studies, 37 were categorized as high quality, 17 as moderate quality and 1 as low quality. Variability was observed mainly in the comparability domain, reflecting differences in adjustment for chronic pelvic pain and other confounding factors.

### 3.1. Psychosocial and Somatic Determinants of the Quality of Life of Women with Endometriosis

Endometriosis symptoms significantly impact patients’ functioning in almost all areas of life. Malenkul, in her systematic review, indicates that women are forced to plan their daily lives around their illness, which contributes to social withdrawal and reduced professional activity [13]. A prospective case–control study involving 2610 women showed that susceptibility to various pain syndromes and their severity negatively impact health-related quality of life (HRQoL), mood, anxiety and depressive symptoms, and work performance [14]. In the study by A. Ramin-Wright, depressive disorders were found in 27.9% of women with endometriosis and in 10.8% of the control group [15]. Increased levels of anxiety and depression also affect adolescents and young women with endometriosis, 33.4% of whom showed moderately severe levels of depressive symptoms [16]. In another study, using the Hospital Anxiety and Depression Scale and the Rosenberg Self-Esteem Scale, it was noted that beliefs about lower self-esteem among childless women correlated with poorer mental health, suggesting a potential direction for psychotherapeutic interventions [17]. Patients also frequently experience fatigue (50.7%) and insomnia (29.2%) [15]. The use of the Piper Fatigue Scale in a cross-sectional study revealed moderate fatigue in one-third and severe fatigue in half of the women studied. Fatigue was significantly associated with increased anxiety and depression, sleep disturbances, poorer sexual functioning, impaired gastrointestinal function, and reduced quality of life (*p* < 0.050) [18]. These symptoms were linked to pelvic pain, which correlated with other symptoms more strongly than the diagnosis of endometriosis itself [19]. Another systematic review and meta-analysis also confirmed these findings [20]. Due to their number, other results valuable for the research review are briefly described and presented in Table 1.

### 3.2. The Relationship Between Endometriosis Symptoms and Sexual Satisfaction and Partner Relationships

Analysis of available research indicates that endometriosis is a significant and independent risk factor for sexual dysfunction and reduced sexual satisfaction. Women with endometriosis are more likely to report pain during intercourse, avoid sexual activity, and have a lower quality of sexual life compared to women without endometriosis [47,48,49,50]. A study conducted in the Lebanese population showed a significant association between endometriosis and decreased sexual satisfaction, assessed using the Sexual Satisfaction Scale for Women (SSS-W). This association is explained by the influence of the cognitive model of negative expectations regarding sexual experiences, which in turn is explained by the central sensitization theory [49]. Central sensitization is a process in which excessive stimulation and persistent overreactivity of the central nervous system can lead to pain independent of peripheral stimuli. It is characterized by changes in the gray matter volume of structures responsible for pain modulation and neurochemical changes within the brain; therefore, it is possible for pain to recur in patients with endometriosis even in the absence of peripheral stimulation [51]. In a survey of 471 women with endometriosis, over 80% reported high levels of stress related to sexual activity and a negative perception of body image, which correlated with greater sexual distress. Self-compassion, in turn, served a protective function [52]. At the same time, research findings highlight the crucial role of emotional and cognitive factors. The severity of symptoms of depression, anxiety, and sexual distress, as well as the way one perceives one’s body and experiences pain, strongly correlates with the level of sexual distress and impaired quality of life. Not only the pain itself, but also its interpretation, fear of pain, and negative expectations of intercourse, exacerbate difficulties in engaging in sexual activity [48,49,53]. A study conducted at two university centers in Germany and Austria, involving 104 heterosexual couples, found strong emotional interdependence between partners. Higher levels of stress, anxiety, and depression in women correlated with greater pain intensity in men, and vice versa. Lower sexual satisfaction and a lack of social support were associated with greater pain severity [54]. Other studies also emphasize the importance of the quality of the partner relationship and social support in women with endometriosis. They show that relationship satisfaction, partner responsiveness, effective coping strategies, and a sense of support may partially mitigate the negative impact of endometriosis pain and symptoms on sexual functioning. Conflict, lack of understanding, and poor communication increase distress and the risk of persistent sexual problems [48,55,56]. Everyone agrees that including your partner in therapy, as well as the importance of emotional support and good communication, are key factors in managing the symptoms of endometriosis.

### 3.3. Potential Mechanisms Linking Endometriosis and Depression

#### The Importance of Pain and Inflammation in Endometriosis

Research indicates that many patients with endometriosis experience a type of pain that may resemble neuropathy. In the screening questionnaire pain DETECT, 40% of the study participants reported pain as neuropathic, while 35% reported mixed neuropathic-nociceptive pain. Patients with a dominant neuropathic component reported more intense pain, increased stress, and cognitive impairment [57]. These are the characteristics often observed in women with EM. Another study found that 79% of people with EM experienced sudden, paroxysmal episodes of pain, distinguishing them from cases of other neuropathic conditions [58].

Chronic menstrual pain leads to changes in the processing of nociceptive stimuli. In patients with dysmenorrhea, untreated pain recurs cyclically, leading to hyperalgesia, a lowered pain threshold, and concomitant hypersensitivity to mild stimuli. Neurotransmitter dysregulation, including excessive glutamate activation, influences the expansion of the nociceptive field and modulates symptoms of dyschezia, dysuria, and dyspareunia [59]. Persistent pain causes tension in the pelvic floor muscles, which in turn worsens pain, dysfunction and sexual problems [60]. Fear of pain can impair relaxation, exacerbating symptoms. Elevated levels of proinflammatory cytokines: IL-6, IL-8, TNF-α, and prostaglandins (PGE2) in patients with endometriosis lead to stimulation of splanchnic nerves, which also leads to increased pain perception. Chronic inflammation with accompanying nociceptor stimulation can lead to central changes, even in the absence of visible morphological changes, increasing the risk of developing chronic pain syndrome [61].

Our analysis of studies across diverse patient populations with EM revealed that chronic pain, not the severity of the disease, is a significant factor influencing women’s mental health. Studies describing various pain phenotypes indicated that specific pain profiles, such as noncyclical, diffuse, or high-intensity pain, are associated with higher levels of depression, anxiety, and greater limitations in social and occupational functioning [62,63,64,65,66].

Many publications have also noted that negative emotions can intensify the experience of pain, leading to a self-reinforcing cycle of interaction between pain symptoms and psychological well-being [67,68,69].

Additionally, numerous analyses have highlighted the importance of factors accompanying pain, such as fatigue, insomnia, low self-esteem, relationship problems and difficulties in sexual functioning, which may perpetuate and strengthen the symptoms of depressive syndrome [53,70,71,72,73].

Some studies have also suggested that chronic pain leaves a long-term impact on emotional functioning, regardless of the current disease state, indicating the possibility of a so-called “lasting pain mark” in patients [74,75].

In summary, the available data suggest that pain, particularly its nature, intensity, and chronicity, is the primary mediator of the relationship between endometriosis and mental disorders. However, the stage of endometriosis plays a secondary role. These results are consistent with previous clinical observations and indicate the need for a holistic therapeutic approach, encompassing both pain management and psychological support for patients with EM.

### 3.4. The Importance of the Gut–Brain Axis in the Pathogenesis of Mental Disorders in Patients with Endometriosis

Growing evidence suggests that alterations in the gut microbiome may play a role in the pathophysiology of endometriosis and its associated psychological burden [76,77]. Women with endometriosis demonstrate differences in gut microbiota composition and diversity, as shown in systematic reviews and meta-analyses, which may contribute to systemic inflammation and altered immune responses [78].

These findings are particularly relevant in the context of the gut–brain axis, a bidirectional communication system linking the gastrointestinal tract and the central nervous system. Dysregulation of this axis may influence emotional processing, stress response, and pain perception. Chronic inflammation and increased intestinal permeability observed in endometriosis may lead to activation of the hypothalamic–pituitary–adrenal (HPA) axis and sustained cortisol elevation, contributing to anxiety and depressive symptoms [79].

Additionally, gut microbiota produces neuroactive compounds such as serotonin, gamma-aminobutyric acid (GABA), and short-chain fatty acids (SCFAs), which are known to influence central nervous system function and emotional regulation [80].

Although the gut–brain axis has been widely studied in general populations, evidence specific to endometriosis remains limited and should be interpreted with caution. Further research is required to clarify causal relationships and underlying mechanisms.

### 3.5. Genetic and Epigenetic Relationships Between Endometriosis and Depression

The link between endometriosis and mood disorders, including depression, is increasingly being discussed, yet it remains poorly understood. In one meta-analysis, genomic analysis incorporating data from UK Biobank and FinnGen showed that the risk of several reproductive disorders, including endometriosis, is partially due to a common genetic factor and is significantly correlated with the risk of depression in women. These results may suggest common biological mechanisms linking endometriosis and other reproductive disorders to the risk of depression. These mechanisms include, among others, disturbances in estrogen signaling; variants in the ESR1 gene simultaneously affect the functioning of endometrial tissues and neuronal sensitivity to estrogen. This also has implications for mood regulation and changes in sex hormone regulation, which are associated with FSHB variants, which modulate the function of the hypothalamic–pituitary–ovarian axis [81]. Research indicates a frequent co-occurrence of endometriosis with psychiatric disorders, especially those with predominant anxiety and depressive symptoms. Women with EM in the study had a higher risk of depression (HR 1.48–1.56) and anxiety disorders (HR 1.38–1.44) [82]. Genetic analyses suggest that this association is not solely due to chronic pain but also has a biological basis. A common genetic etiology for endometriosis and depression has also been demonstrated, as well as the existence of common gene loci associated with these conditions [83].

The review of genetic analyses also included studies using Mendelian randomization (MRA), a genetic epidemiology method based on randomly inherited genetic variants. This approach allows for a rough estimation of possible causal relationships, but interpretation requires caution due to the risk of pleiotropy and the influence of confounding factors. The results indicate that genetic susceptibility to depression may be associated with an increased risk of endometriosis (OR ≈ 1.25) in some MRA analyses. However, the authors emphasize that due to the limitations of the method and the complexity of the endometriosis phenotype, these data cannot be considered unequivocal evidence of causality. Current evidence from systematic reviews and meta-analyses suggests that both conditions may share certain genetic components, which may partially explain their frequent co-occurrence [84].

### 3.6. Treatment Strategies and Psychotherapeutic Interventions

Currently, there is no effective causal treatment for endometriosis, and therapeutic methods largely focus solely on surgical removal of endometrial lesions, which is associated with a risk of disease recurrence of up to 40–50% over five years [85]. Symptomatic therapy includes gonadotropin-releasing hormone (GnRH) analogues, danazol, an androgen derivative, and anti-inflammatory drugs, without taking into account the potential neuropathic mechanisms of chronic pain [57,85].

An analysis of five studies found that cognitive behavioral therapy (CBT) provides long-term benefits for women with endometriosis and chronic pelvic pain. The use of psychoeducation and stress management techniques reduces pain, depressive symptoms, and stress levels, resulting in improved quality of life and psychological well-being. These results support the effectiveness of CBT as an adjunct therapy in alleviating both the physical and emotional symptoms of endometriosis [86].

### 3.7. Comparison of the Effectiveness of Psychological Interventions, Partner Role and Self-Acceptance in Patients with Endometriosis

Analysis of research findings on psychological interventions such as cognitive behavioral therapy, self-acceptance training, and couples therapy for women with endometriosis and their partners indicates that emotional support and these interventions significantly improve mental health and relationship functioning. Early studies have already shown that improved relationship quality is associated with reduced stress and better adaptation to disease symptoms [87]. Couples interventions increase relationship cohesion and reduce the psychological burden of pain [54].

Qualitative studies with a smaller sample size confirm that emotional support and education about the disease increase the sense of control over symptoms [70], and regular work with a psychologist reduces emotional tension [88]. In turn, analyses of large groups of patients (from several hundred to over eight hundred) indicate that psychological interventions alleviate symptoms of depression, anxiety, reduced quality of sexual life, and feelings of stigmatization [89,90,91].

One randomized trial showed that an intervention based on environmental enrichment (EE), which is structured, multifaceted stimuli that increase cognitive, sensory, and social activity, significantly reduced symptoms of anxiety and depression and improved quality of life while reducing stress levels in women with endometriosis. Compared to the control group, EE program participants scored lower on depression scales (including the HADS-7) both at the end of therapy and at a three-month follow-up. It was proven that the therapy improves self-esteem, reduces depression levels and increases the sense of empowerment [92]. Similar effects have been observed in studies on large populations, which emphasize the impact of therapy on reducing anxiety and improving quality of life [93]. A three-month program based on cognitive behavioral therapy and quality of life indicators, developed by Z. Breton et al., demonstrated that multidisciplinary self-management tools can be helpful in reducing symptoms, anxiety, and neuropathic pain [43].

## 4. Discussion

Endometriosis is a disease that should be considered from a multidimensional perspective and continues to pose a significant clinical problem. Its consequences extend beyond the reproductive system and, as evidenced by the data cited above, also affect the psychological and social spheres [8,11,13,23,24,38]. Chronic pelvic pain—one of the main symptoms of endometriosis—correlates with higher levels of anxiety and depression, poorer sleep quality, sexual dysfunction, and reduced quality of life [9,19,20,27,64]. A prolonged time from symptom onset to diagnosis further increases psychological burden and worsens prognosis [13,14,29,33,41]. The coexistence of endometriosis and mental disorders is multifactorial. It includes biological factors (chronic inflammation, oxidative stress, genetic and epigenetic conditions) [6,61,78,81,84], psychological factors (pain catastrophization, anticipatory anxiety) [21,53,63,73], and social factors (stigma, social isolation) [40,44,45]. Increasing evidence also indicates a partially common biological basis for endometriosis and depression [81,82,83,84]. An additional factor worsening the quality of life of women with EM is sexual dysfunction, which negatively impacts partner relationships and the emotional well-being of patients [49,50,54]. Therefore, it is crucial to implement integrated care models encompassing not only surgical and pharmacological treatment but also psychological and psychosocial interventions [11,23,38]. Early identification of anxiety and depressive symptoms and access to interdisciplinary care can significantly improve prognosis, reduce pain intensity, and increase patients’ quality of life [86,89,91,93]. Although not included in this review due to strict exclusion criteria aimed at minimizing confounding, obstructive sleep apnea (OSA) may represent a clinically relevant comorbidity in women with endometriosis. Given its established association with mental health outcomes, future studies should explore the potential role of sleep-disordered breathing in this population [94].

This study has several strengths. First, a comprehensive and systematic search strategy was applied across multiple databases, ensuring broad coverage of the available literature. Second, the inclusion of a large number of studies enhanced the robustness and generalizability of the findings. Third, efforts were made to minimize bias by applying predefined inclusion and exclusion criteria, and the evaluation of scientific papers was done using the Newcastle-Ottawa scale.

However, several limitations should be acknowledged. The study was not registered in PROSPERO, which may increase the risk of selection bias. The included studies were heterogeneous in terms of design, population characteristics, and outcome measures, which may affect the comparability of results. Additionally, most studies were observational, limiting the ability to infer causality, and the quality of included studies varied. The exclusion of studies involving comorbid conditions, while necessary to reduce confounding, may limit the generalizability of the findings to real-world clinical populations. Despite these limitations, the findings provide valuable insight into the relationship between endometriosis and mental health.

## 5. Conclusions

The review findings emphasize the need for an integrated therapeutic approach, combining medical treatment with psychological and educational interventions, and elements of social support. Cognitive-behavioral therapies, self-care programs, couples work, and techniques that enhance self-acceptance can significantly reduce the severity of psychopathological symptoms and improve quality of life and relationship functioning in women with endometriosis. Table 2 summarizes the health and psychosocial consequences of endometriosis.

## Figures and Tables

**Table 1:** Characteristics of other studies on quality of life, depression and anxiety in patients with endometriosis.

Position	Type of Psychosocial Factor	Study Type, Study Group Size	Methodology	Conclusions	Source
1	Affective; Cognitive	Systematic review N = 5419	NRS, VAS, SF-MPQ, HRQoL (EHP-30, SF-36), HADS, BDI/BAI, PSS, PCS, PSWQ, DERS, TAS, AIS, Rosenberg Self-Esteem, personality scales	Support, biopsychosocial model of endometriosis and highlighting the role of psychosocial factors in the development of the disease.	Kalfas M, Chisari C, Windgassen S. Psychosocial factors associated with pain and health-related quality of life in endometriosis: a systematic review. Eur J Pain.2022;26(10):2070–2088. [20]
2	Affective; Social	Cross-sectional + observation al study N = 205	FPI, EHP-30, BDI	The impact of somatic and emotional symptoms on quality of life and psychosocial burden.	Matasariu RD, Onofriescu M, Ionescu CA, et al. Psycho-social aspects of quality of life in women with endometriosis. Acta Endocrinol (Buchar). 2017;13(3):334–339. [21]
3	Affective; Social; Cognitive/Behavioral	Narrative Review	Depression and anxiety scales; HRQoL (EHP-30, SF-36); pain scales	The adverse impact of anxiety, depression, stress and social isolation on quality of life regardless of the stage of endometriosis.	Dipankar SP, Sharma S, Patel R, et al. Psychological distress and quality of life in women with endometriosis: a narrative review of therapeutic approaches and challenges. Cureus. 2025;17(1):e80180. [22]
4	Affective	Systematic review (34 studies), meta-analysis (15 studies)	HADS, BDI, STAI; diagnostic tools for mental disorders; CPP; dyspareunia; HRQoL	Endometriosis is a condition associated with significant psychological burden, including anxiety and depression.	Delanerolle G, Niazi A, Lu Z, et al. A systematic review and meta-analysis of endometriosis and mental health sequelae: the ELEMI project. Womens Health (Lond). 2021;17:17455065211019717. [23]
5	Affective	Cross-sectional study N = 514	Pelvic pain questionnaire (NRS); EQ-5D, EQ-VAS; Ultrasound according to IDEA	Deep or ovarian endometriosis visualized on ultrasound does not always correlate with pain and quality of life, which justifies the diagnosis of other causes of the symptoms and the importance of comprehensive counseling.	Chaggar P, Alcazar JL, Guerriero S, et al. Impact of deep or ovarian endometriosis on pelvic pain and quality of life: a prospective cross-sectional ultrasound study. Ultrasound Obstet Gynecol. 2025;65(3):372– 383. [24]
6	Affective; Cognitive/Emotional	Cross-sectional study N = 338	ISI, HADS, DERS-SF; sleep measures (satisfaction, vigilance, efficiency, timing)	Bidirectional relationship between pain and anxiety symptoms and the relationship between sleep quality and the severity of endometriosis and psychological difficulties	Baldi E, Meneo D, et al. Sleep health and psychological well-being in adult women: a specific focus on endometriosis—a survey study. J Clin Med. 2025;14(6):2103. [25]
7	Affective; Cognitive/Social	Cross-sectional observation al study N = 139	SF-12 (PCS, MCS), HAMA, SDS	Strong association between quality of life and anxiety levels and the presence of depression in women with endometriosis.	He G, Chen J, Peng Z, Luo C, Zeng X. Correlation between quality of life and unhealthy emotions among patients with endometriosis. Front Psychol. 2022;13:830698. [26]
8	Affective; Behavioral/Emotion Regulation	Case-control study (matched pair) N = 246	PSQI, ISI, ESS, HADS, SF-12	The key importance of sleep and fatigue in the comprehensive care of women with endometriosis.	Facchin F, Burggio L, Roncella E, et al. Sleep disturbances, fatigue and psychological health in women with endometriosis: a matched pair case-control study. Reprod Biomed Online. 2021;43(6):1035–1044. [18]
9	Affective; Behavioral/Emotion Regulation	Cross-sectional study N = 230	PFS, HADS, PCS, PSQI, GIQLI, FSFI, MOS-SSS, EHP-30	Fatigue as a key symptom of endometriosis, mediating the relationship between pain, psychosocial functioning and quality of life, indicating the need to consider fatigue and cognitive factors in planning interventions.	Mundo-López A, Carmona F, Gómez-Carranza E, et al. Contribution of chronic fatigue to psychosocial status and quality of life in Spanish women diagnosed with endometriosis. Int J Environ Res Public Health. 2020;17(11):3831. [17]
10	Affective; Social	Cross-sectional study (case-control/population) N = 2610	SF-36v2, WPAI, WERF-EPHect EPQ-M	Significant deterioration in quality of life and productivity in Arab women with endometriosis.	Mousa M, Al-Jefout M, Alsafar B, Becker CM, Zondervan KT, Rahmioglu N. Impact of endometriosis in women of Arab ancestry on health-related quality of life, work productivity, and diagnostic delay. Front Glob Womens Health. 2021;2:708410. [13]
11	Affective; Social; Cognitive/Behavioral	Cross-sectional study (Mixed-methods) N = 23	HADS, SF-12, PHQ-15, Functional Well-being-7; interviews; symptom diary	Significant association of anxiety and depression with endometriosis in research results.	Olliges E, Bobinger A, Weber A, et al. Physical, psychological, and social day-to-day experience of women living with endometriosis compared to healthy age-matched controls: a mixed-methods study. Front Glob Womens Health. 2021;2:767114. [27]
12	Affective; Social; Somatic	Cross-sectional/an alytical study N = 953	QoL tools: EHP-30, SF-36; registry/survey data	The persistent strong impact of endometriosis on quality of life in the general population; the need to include the problem in clinical care also outside specialist centres.	Kogelman LJK, et al. The burden of endometriosis on quality of life in Danish women: an analysis of the Danish Blood Donor Study. BMC Med. 2025;23. [28]
13	Affective; Somatic	Cross-sectional study N = 199	SHAPS, BDI-II, HADS, NRS	The need to expand care for patients with endometriosis to include the assessment of anhedonia and loss of pleasure, in addition to symptoms of anxiety and depression.	Mallorquí A, Fortuna A, Segura M, et al. Prevalence of anhedonia in women with deep endometriosis. Sci Rep. 2024;14:84772. [29]
14	Behavioral; Social; Somatic	Cross-sectional study N = 200	HPLP, EIQ	The need to integrate health-promoting activities and support a healthy lifestyle in endometriosis treatment strategies.	Mollazadeh S, Najmabadi KM, Mirghafourvand M. Health-promoting lifestyle and its relationship with the effects of endometriosis on women’s lives in Iran: a cross-sectional study. BMC Womens Health. 2022;22. [30]
15	Cognitive; Affective; Somatic	Narrative Review	SF-36, WHOQOL-BREF, EQ-5D, EHP-30, EHP-5, FertiQoL, FPI	A review of QoL tools confirming the need for a multidimensional approach, encompassing psychological well-being and social functioning.	Manu A. Quality of life assessment and clinical implications for women with endometriosis through validated tools: a narrative review. Medicina (Kaunas). 2024;61(10):1729. [31]
16	Affective; Cognitive; Somatic	Cross-sectional study N = 653	DASS-21, EHP-5, NRS	Diagnostic delays as a significant source of psychological burden; the need for therapeutic support before diagnosis.	Mosterd D, et al. Comparing psychological distress, health-related quality of life and pain in diagnosed versus suspected endometriosis. Preprint. 2025. [32]
17	Behavioral; Affective; Somatic	Randomize d controlled trial N = 31	PFS, PSQI, HADS, GIQLI, FSFI; physical fitness tests: back dynamometer, Schöber, Flamingo, 6MWT	Multimodal therapeutic training as an effective support for the treatment of endometriosis.	Salinas-Asensio M.D.M., et al. Changes in fatigue, sleep quality, mental health and sexual function after a multimodal supervised exercise program in women with endometriosis unresponsive to conventional therapy. Eur J Obstet Gynecol Reprod Biol. 2025;114083. [33]
18	Cognitive; Affective; Behavioral; Social	Cross-sectional study N = 262	PPIQ, PSEQ, PSS-10, NRS, GPAQ, SF-36	Results supporting a biopsychosocial approach to endometriosis.	Kovács-Szabó Z, Ács P, Prémusz V, Makai A, Hock M. Psychological, symptom-related, and lifestyle predictors of health-related quality of life in Hungarian women with endometriosis. J Clin Med. 2021;10:7004. [34]
19	Affective; Cognitive	Cross-sectional study N = 425	EHP, LOT-R, GSES; socio- Economic and diagnostic-therapeutic questionnaire	Self-efficacy and optimism as protective psychological resources in endometriosis; need for integration in clinical care.	Bień A, Pokropska A, Grzesik-Gąsior J, et al. Quality of life in women with endometriosis: the importance of sociodemographic, diagnostic-therapeutic, and psychological factors. J Clin Med. 2025;14(12):4268. (Bień, Pokropska, Grzesik-Gąsior, Korżyńska-Piętas, Pieczykolan Agnieszkaand Zarajczyk, et al., 2025) [35]
20	Behavioral; Social; Somatic	Cross-sectional study N = 200	HPLP, EIQ; sociodemograph ic questionnaires	The need to take into account lifestyle factors and social support in carepatientswith endometriosis.	Mollazadeh S., Najmabadi KM, Mirghafourvand M. Health-promoting lifestyle and its relationship with the effects of endometriosis on women’s lives in Iran: a cross-sectional study. BMC Womens Health. 2022;22. [30]
21	Cognitive; Behavioral; Affective	Cross-sectional/an alytical study N = 644	QoL clustering; SF-36 or other QoL profile	Personalized care as a key element in the management of women with endometriosis.	Vallée A. Quality of life identification by unsupervised cluster analysis: a new approach to modeling the burden of endometriosis. PLoS One. 2024;19:e0317178. [36]
22	Affective; Cognitive; Behavioral; Social	Narrative literature review	EHP-30, SF-36, HADS, BDI; pain scales; biopsychosocial models	The need to implement a biopsychosocial approach in clinical care.	Noditi A., et al. Analysis of the biopsychosocial impacts associated with endometriosis to improve patient care. J Clin Med. 2023;12(7):2158. [37]
23	Affective; Cognitive; Behavioral	Narrative literature review	HADS, EHP-30; adolescent health tools	Early diagnosis and psychological support as key elements of care; significant impact of adolescent endometriosis on psychosocial development.	Panvino F., et al. Endometriosis in adolescence: A narrative review of the psychological and clinical implications 2025;15(5):548. [38]
24	Affective; Somatic	Cross-sectional study N = 212	SHAPS, NRS, HADS, BDI-II	Anhedonia as an overlooked aspect of mental health; an indication for routine psychological assessment in women with endometriosis.	Mallorquí A, Fortuna A, Segura M, et al. Prevalence of anhedonia in women with deep endometriosis. Sci Rep. 2024;14:84772. [29]
25	Affective; Social; Cognitive	Cross-sectional study (Mixed-methods) N = 533	FSI, DASS-21, MSPSS, WHOQOL-BREF	Validation of patient experiences as one of the key determinants of quality of life	Katz C, Armor M, et al. “Listen to women as if they were your most cherished person”: an Australian mixed-methods study on endometriosis care. J Health Psychol. 2024;29:13591053241250101. [39]
26	Affective; Cognitive; Social	Prospective cohort study N = 205	EHP, VAS, EHP-30	No evidence of improved quality of life as a result of early diagnosis and treatment; confirmed pain reduction in patients.	Kaveh M, Nakhaee Moghadam M. The impact of early diagnosis of endometriosis on quality of life. Arch Gynecol Obstet. 2025;312. [40]
27	Affective; Cognitive; Symptom-related	Prospective study (quasi- experimenta l) N = 117	SF-36, STAI-Y1, SDS, VAS	Pain reduction does not necessarily translate into improved mental health.	Cagnacci A, et al. Chronic pelvic pain improvement: impact on quality of life and mood. J Psychosom Obstet Gynaecol. 2018;39(4):256–262. [41]
28	Cognitive; Behavioral; Affective	Pilot cohort study (digital health) N = 241	Symptom Monitoring App; EHP-30; NRS; EHP-5; EQ-5D	Digital programs as a valuable addition to therapy for women with endometriosis.	Breton Z, Stern E, et al. A digital program for daily life management with endometriosis: a feasibility study. JMIR. 2023;25:e58262. [42]
29	Social; Affective; Cognitive	Cross-sectional study (mixed-methods) N = 389	EHP-30, VIRS, PHQ-9, GAD-7	The key role of communication in shaping the quality of life; validation of the patient’s experience as a protective factor, lackvalidation as a risk factor for worsening HRQoL.	Grundström H, et al. Experiences of communication with healthcare professionals in women with endometriosis. Health Commun. 2023. [43]
30	Social; Affective; Cognitive	Cross-sectional study N = 543	Social Negativity Scale, PLCI, SF-12	The negative impact of social relationships as a psychosocial factor; the importance of assessing patients’ social environment and psychoeducational interventions.	Zarecki C. et al. Understanding the role of social negativity in percived life course impact and mental health among woman with endometriosis. J Clin Med. 2025;14(13):4761. [44]
31	Affective; Cognitive; Symptom-related	Cross-sectional study with regression N = 175	EHP-30, NRS, VAS, ESHRE guidelines	Quality of life depends on the nature of the symptomsmorethan the stage of disease progression; the crucial importance of pain control.	Kupec T., et al. The multifactorial burden of endometriosis: prediction of quality of life. J Clin Med. 2025;14(2):323. [45]
32	Affective; Symptom-related	Cross-sectional study N = 425	EHP-30, VAS/NRS, symptom scales	The importance of effective pain management and consideration of comorbidities for improving QoL.	Bień A., et al. Clinical factors affecting the quality of life of women with endometriosis. J Adv Nurs. 2025;81(8):4667–4680 [46]

Note: AIS—Athens Insomnia Scale, BAI—Beck Anxiety Inventory, BDI/BDI-II—Beck Depression Inventory./Beck Depression Inventory-II, CPP—Chronic Pelvic Pain, DASS-21—Depression, Anxiety and Stress Scale-21, DERS/DERS-SF—Difficulties in Emotion Regulation Scale/Short Form, EHP/EHP-30/EHP-5—Endometriosis Health Profile/30-item/5-item, EIQ—Endometriosis Impact Questionn, EQ-5D/EQ-VAS—EuroQol 5 Dimensions/Visual Analogue Scale, EPQ-M—EPHect Minimum Clinical Questionnaire, ESS—Epworth Sleepiness Scale, FertiQoL—Fertility Quality of Life, FPI—Fertility Problem Inventory, FSFI—Female Sexual Function Index, FSI—Fatigue Symptom Inventory, GAD-7—Generalized Anxiety Disorder-7, GIQLI—Gastrointestinal Quality of Life Index, GPAQ—Global Physical Activity Questionnaire, GSES—General Self-Efficacy Scale, HADS—Hospital Anxiety and Depression Scale, HAMA—Hamilton Anxiety Rating Scale, HPLP—Health-Promoting Lifestyle Profile, HRQoL—Health-Related Quality of Life, ISI—Insomnia Severity Index, LOT-R—Life Orientation Test-Revised, MOS-SSS—Medical Outcomes Study Social Support Survey, MSPSS—Multidimensional Scale of Perceived Social Support, NRS—Numeric Rating Scale, PCS—Pain Catastrophizing Scale, PHQ-9/PHQ-15—Patient Health Questionnaire-9/Somatic Symptoms-15, PLCI—Perceived Life Course Impact, PPIQ—Pelvic Pain Impact Questionnaire, PFS—Piper Fatigue Scale, PSEQ—Pain Self-Efficacy Questionnaire, PSQI—Pittsburgh Sleep Quality Index, PSS/PSS-10—Perceived Stress Scale/10-item, PSWQ—Penn State Worry Questionnaire, SDS—Self-Rating Depression Scale, SF-12/SF-36/SF-36v2—Short Form Health Survey, SHAPS—Snaith–Hamilton Pleasure Scale, STAI/STAI-Y1—State-Trait Anxiety Inventory/Form Y-1, TAS—Toronto Alexithymia Scale, VAS—Visual Analogue Scale, VIRS—Validation and Invalidation Response Scale, WPAI—Work Productivity and Activity Impairment, WERF-EPHect—World Endometriosis Research Foundation Endometriosis Phenome and Biobanking Harmonization Project, WHOQOL-BREF—World Health Organization Quality of Life-BREF.

**Table 2:** Health and psychosocial consequences of endometriosis.

Somatic Symptoms and Complications	Impact on Mental Well-Being	Important Psychological Factors	Psychosocial Consequences
Chronic pelvic pain	Depressive disorders, low mood, anxiety	Pain catastrophization, anticipatory anxiety, feeling of helplessness	Lack of understanding from the environment, diagnostic delay, lack of adequate support
Painful periods (dysmenorrhoea)	Emotional tension, irritability	Reduced stress tolerance, negative beliefs about the disease	Professional withdrawal, marginalization of symptoms (“normalization of pain”)
Abnormal bleeding	Sleep disturbances, exhaustion	Constant worrying	Difficulties in fulfilling professional roles and social activities
Dyspareunia	Decreased quality of sexual life, avoidance of intercourse	Fear of pain, negative body image	Tensions in the relationship, decreased satisfaction with the partner relationship
Pain when urinating/defecating	Intensification of somatic symptoms of anxiety	Interoceptive hypersensitivity, avoidance of painful stimuli	Social isolation
Fatigue, insomnia	Increased susceptibility to depressive and anxiety disorders	Emotional regulation disorders, low mental resilience	Deterioration of productivity in every sphere of functioning
Fertility problems	Sense of loss, lowered self-esteem	Internalization of negative beliefs about oneself	Stigmatization, feeling of social pressure, relationship tensions

## Data Availability

The datasets analyzed during the current study are available in the primary research articles cited in the References section.

## Supplementary Materials

The following supporting information can be downloaded at https://www.mdpi.com/article/10.3390/jcm15103598/s1, Table S1. Newcastle–Ottawa Scale (NOS), Table S2. Newcastle–Ottawa Scale (NOS) adapted for cross-sectional studies, and PRISMA checklist [95,96].

## Abbreviations

CBT	Cognitive Behavioral Therapy
CNS	Central Nervous System
DETECT	Neuropathic Pain DETECT Questionnaire
EE	Environmental Enrichment
EM	Endometriosis
GABA	Gamma-Aminobutyric Acid
GnRH	Gonadotropin-Releasing Hormone
HADS-7	Hospital Anxiety and Depression Scale-7
HPA	Hypothalamic–Pituitary–Adrenal
HRQoL	Health-Related Quality of Life
IL-6	Interleukin 6
IL-8	Interleukin 8
MeSH	Medical Subject Headings
MRA	Mendelian Randomization Analysis
OR	Odds Ratio
PGE2	Prostaglandin E2
PMC	PubMed Central
PRISMA	Preferred Reporting Items for Systematic Reviews and Meta-Analyses
PROSPERO	International Prospective Register of Systematic Reviews
SCFAs	Short-Chain Fatty Acids
SSS-W	Sexual Satisfaction Scale for Women
TNF-α	Tumor Necrosis Factor Alpha
UK	United Kingdom
WHO	World Health Organization
